# Sesquiterpene Lactones Attenuate Paclitaxel Resistance Via Inhibiting MALAT1/STAT3/ FUT4 Axis and P-Glycoprotein Transporters in Lung Cancer Cells

**DOI:** 10.3389/fphar.2022.795613

**Published:** 2022-02-24

**Authors:** Yaming Ding, Zhang Zhen, Muhammad Azhar Nisar, Farman Ali, Riaz Ud Din, Muhammad Khan, Tafail Akbar Mughal, Gulzar Alam, Linlin Liu, Muhammad Zubair Saleem

**Affiliations:** ^1^ The Second Hospital of Jilin University, Changchun, China; ^2^ College of Basic Medical Sciences, Dalian Medical University, Dalian, China; ^3^ Academy of Integrative Medicine, Fujian University of Traditional Chinese Medicine, Fuzhou, China; ^4^ Institute of Zoology, University of the Punjab, Lahore, Pakistan; ^5^ Medical Toxicology Laboratory, Department of Zoology, Women University of Azad Jammu and Kashmir, Muzaffarabad, Pakistan; ^6^ Faculty of Rehabilitation and Allied Health Sciences, Riphah International University, Islamabad, Pakistan; ^7^ Fujian Provincial Key Laboratory of Natural Medicine Pharmacology, School of Pharmacy, Fujian Medical University, Fuzhou, China

**Keywords:** paclitaxel resistance, STAT3, FUT4, P-gp, MALAT1, alantolactone, brevilin A

## Abstract

Paclitaxel resistance is a challenging factor in chemotherapy resulting in poor prognosis and cancer recurrence. Signal transducer and activator of transcription factor 3 (STAT3), a key transcription factor, performs a critical role in cancer development, cell survival and chemoresistance, while its inactivation overwhelms drug resistance in numerous cancer types including lung cancer. Additionally, the fucosyltransferase 4 (FUT4) is a crucial enzyme in post-translational modification of cell-surface proteins involved in various pathological conditions such as tumor multidrug resistance (MDR). The P-glycoprotein (P-GP) is the well-known ABC transporter member that imparts drug resistance in different cancer types, most notably paclitaxel resistance in lung cancer cells. LncRNA-MALAT1 exerts a functional role in the cancer development as well as the drug resistance and is linked with STAT3 activation and activity of FUT4. Moreover, STAT3-mediated induction of P-GP is well-documented. Natural compounds of Sesquiterpene Lactone (SL) family are well-known for their anticancer properties with particular emphasis over STAT3 inhibitory capabilities. In this study, we explored the positive correlation of MALAT1 with STAT3 and FUT4 activity in paclitaxel resistant A549 (A549/T) lung cancer cells. Additionally, we investigated the anticancer activity of two well-known members of SLs, alantolactone (ALT) and Brevilin A (Brv-A), in A549/T lung cancer cells. ALT and Brv-A induced apoptosis in A549/T cells. Furthermore, these two natural SLs suppressed MALAT1 expression, STAT3 activation, and FUT4 and P-GP expression which are the hallmarks for paclitaxel resistance in A549 lung cancer cells. The inhibition of MALAT1 enhanced the competence of these SLs members significantly, which accounted for the growth inhibition as well as anti-migratory and anti-invasive effects of ALT and Brv-A. These findings suggest SLs to be the promising agents for overcoming paclitaxel resistance in A549 lung cancer cells.

## Introduction

Among different types of cancer, the most lethal type is the lung cancer worldwide having highest mortality, morbidity, and metastatic rate. The most common malignant lung cancer is the non-small cell lung cancer (NSCLC) accounting approximately 90% of all type lung cancer cases whereas 70–80% of patients suffer with an intricate disease state and are unable to undergo the surgical removal of tumor (Wakelee et al., 2014; Siegel et al., 2017; Gong et al., 2018; Teng et al., 2019; Lin et al., 2020). Also, the side effects and drug tolerance in chemotherapy at the advanced stage are overwhelming factors. Due to the drug tolerance, patients within advanced stage of tumor have less response to the chemotherapy (Scheff and Schneider, 2013; Zhou et al., 2019). Paclitaxel is widely used in cancer treatment, primarily as a first-line chemotherapeutic drug against clinical manifestations of NSCLC (Pilkington et al., 2015; Yuan H. et al., 2016; Chen et al., 2017). Although, paclitaxel has improved the curative rates in both earlier and advanced stages of the disease and enhanced furtherance in the quality and life span of NSCLC patients (Zeng et al., 2015), however, paclitaxel resistance is a challenging obstacle to the successful treatment that creates a hiatus in the multimodal chemotherapeutic approach (Suda and Mitsudomi, 2015; Li et al., 2019).

Chemoresistance is a result of a variety of mechanisms that aid cancer cells in overcoming stress conditions. Previous studies have revealed that genetic and epigenetic modifications can lead to both acquired and intrinsic chemoresistance (Rebucci and Michiels, 2013). The long non-coding RNAs (lncRNAs) are the type of non-coding RNA transcripts containing more than 200 nucleotides that lack or have a limited protein-coding potential (Qu et al., 2016). It is known that lncRNAs perform important role in transcriptional and post-transcriptional regulation, chromatin remodeling, microRNA sponging, and chemoresistance in cancer biology (YiRen et al., 2017; Kim et al., 2020). MALAT1 (Metastasis-associated lung adenocarcinoma transcript 1), primarily discovered in NSCLC, is evolutionarily conserved lncRNA located at chromosome 11q13 that plays a variety of biological functions, i.e., glycolysis, vascular growth, retinal neurodegeneration, cancer progression, and chemotherapeutic resistance (Michalik et al., 2014; Latorre et al., 2016; Luo et al., 2016; Yao et al., 2016).

The increased levels of drug resistance-associated transporters encoded by ATP-binding cassette (ABC) genes in lung cancer are the most significant contributors to drug resistance (Zheng et al., 2021). Among seven sub-families, 13 out of 49 members of ABC genes are implicated in drug resistance. Among these, MDR1, also identified as P-GP (P-glycoprotein) plays a key role in the transportation or inhibition of 324 approved drugs and is known to ease the paclitaxel transport across the membrane (Nanayakkara et al., 2018). STAT3 is an imperative transcription factor involves in the regulation of MDR1 gene expression at transcriptional level. Recent studies have demonstrated that MALAT1 contributes to drug resistance by modulating STAT3 activation. STAT3 has also been implicated in cisplatin, doxorubicin, and gefitinib resistance (Phan et al., 2016; Fang et al., 2018; Li et al., 2018; Yue et al., 2020; Cao et al., 2021). Furthermore, STAT3 inactivation incapacitates paclitaxel resistance in several carcinomas as well as lung cancer in either independent or synergistic manner (Lee et al., 2015; Ji et al., 2017). Therefore, inactivating STAT3 *via* STAT3 inhibitors is a significant approach to devastate the resistance of paclitaxel in lung cancer.

Fucosyltransferase (FUT) family is involved in the post-translational modification of proteins by catalyzing the transfer of fucose residue to acceptor of oligosaccharide from the donor substrate GDP-fuc in various linkages, i.e., a1, 2-(FUT2 and FUT1), a1, 3/4-(FUT4, FUT3, FUT6, FUT5, FUT7, FUT10, FUT9, and FUT11) and a1, 6-linkage (FUT8) (de Vries et al., 2001; Ma et al., 2006; Cheng et al., 2013). The implications of these fucosylated oligosaccharides are noticed in different biological processes that include cell-cell interactions in differentiation, cancer development, and tumor multidrug resistance. In previous studies, FUT4 has been associated in cancer development and multidrug resistance *via* PI3K/AKT signaling pathway. Moreover, FUT4 regulation has also been elaborated in colorectal cancer where miR-26a/26b is sponged by MALAT1 to enhance the FUT4 fucosylation and promotes the metastasis *via* activation of PI3K/AKT pathway (Xu et al., 2020). Therefore, STAT3 activation and glycan alterations are the promising therapeutic targets to overcoming multidrug resistance in cancer.

Sesquiterpene lactones (SLs) are secondary metabolites comprise over 5,000 known compounds found in various plant families such as Cactaceae, Euphorbiaceae, Solanaceae, and Araceae (Wedge et al., 2000; Canales et al., 2005). These are commonly found in Asteraceae family which are one of the diverse plants in the world. SLs account up a significant portion of dry mass of these plants i.e., 3% in *Helenium amarum* (Ivie et al., 1975). SLs are colorless, lipophilic molecule with a 15-carbon backbone chain. To accomplish the goals, we used previously reported STAT3 inhibitors and members of SLs family, Alantolactone (ALT) and Brevilin A (Brv-A), based on their current effectiveness against multiple cancer cell lines. In previous study, the effect of ALT is investigated to reduce resistance of doxorubicin in A549 lung cancer cells *via* targeting MDR1 and STAT3 (Maryam et al., 2017). Beside, we have reported that Brv-A inhibits the activation of STAT3 even more significantly than the commercially available STAT3 inhibitors by direct binding (Khan et al., 2020; Saleem et al., 2020). This study represents the key role of these two compounds in reducing the expression of FUT4 in paclitaxel-resistant A549 (A549/T) cells for the first time. Several studies are previously conducted to discover the role of lncRNA MALAT1 in chemoresistance, however, how MALAT1 functions in the context of a therapeutic approach with special prominence to MALAT1/STAT3 and MALAT1/FUT4 axis in paclitaxel-resistant lung cancer is not well-known. Therefore, the feedback role of MALAT1 during STAT3 inhibition and FUT4 is needed to be justified analytically. The current work sought to provide novel insights into the functional response of MALAT1 and efficacy of common members of SLs (ALT and Brv-A) in paclitaxel-resistant lung cancer, vis-à-vis MALAT1/STAT3 and MALAT1/FUT4 axis.

## Materials and Methods

### Reagents and Chemicals

Alantolactone (ALT) (>98% purity) was obtained from Tauto-Biotech (Shanghai, China) while Brevilin A (Brv-A) (purity >98%) and Paclitaxel (PTX) (purity >99%) was obtained from Selleck (China). DMEM (Dulbecco’s modified Eagle’s Medium), trypsin without or with EDTA, Streptomycin-Penicillin (10,000 U/ml), and fetal bovine serum (FBS) were all purchased from Gibco (Thermo Fisher Scientific, Inc.). Cell counting kit-8 (CCK-8) was purchased from KeyGen Biotech (Nanjing, China) and Protease-inhibitor cocktail from Sigma-Aldrich (St. Louis, MO). Dimethyl Sulfoxide (DMSO), Phenyl methyl sulfonyl fluoride (PMSF), Propidium Iodide (PI), crystal violet stain, Calcein acetoxymethyl ester (Calcein-AM), and annexin V-FITC kit for apoptosis detection were obtained from Beyotime Biotechnology (China). The primary antibodies for cleaved caspase-3, cleaved caspase-9 and cleaved PARP as well as p-STAT3, and STAT3 (Tyr 705) were obtained from Cell-Signaling Technology (Beverly, MA). FUT4 from Proteintech (Wuhan, China) while P-GP was obtained from abcam (Cambridge, MA). GAPDH was procured from Beyotime Biotechnology (China), while the HRP (Horseradish Peroxidase)-conjugated secondary type of antibodies (anti-mouse and anti-rabbit) were obtained from Sigma-Aldrich (St. Louis, MO).

### Cell Culture

Human non-small cell lung cancer cell line (A549), and paclitaxel-resistant A549 (A549/T) cell line was purchased from iCell Biosciences Inc. (Shanghai, China). Both A549/T and A549 cell lines were cultured in 10% FBS containing DMEM, accompanied 100 μg/ml streptomycin and 100 U/ml concentration of penicillin. The cell lines were maintained in a logarithmic phase of growth for all experiments in humidified atmosphere with 5% CO_2_ at 37°C.

### Cell Treatment and Cell Viability Assay

DMSO (0.5% final concentration) was used to dissolve ALT, Brv-A, and PTX while the same DMSO concentration was kept as a control. CCK-8 kit was used to find out the percentage of cells viability. Briefly, cells (with density of 3 × 10^3^) were added in 96-well culture plates for 24 h and treated with different concentrations of ALT and Brv-A (2.5, 5, 10, 15, 20, 40, 80 and 120 μM) for 24, 48, and 72 h, while various concentrations of PTX (0.0125, 0.25, 0.5, 1, 2, 4, 8, and 16 μM) were used to check the cell viability at 24, 48, and 72 h in A549 and A549/T lung cancer cells. The resistance index was calculated at 72 h time point. Afterwards, CCK-8 solution (10 μl) was added to each well and 96-well culture plate was kept at 37°C for 3 h. Optical density was measured at 450 nm wavelength through fluorescence-microplate reader (Synergy neo HTS multimode-microplate reader, BioTek). Following formula was used to calculate the cell viability in experiments performed in triplicate.

Cell viability (%)=(A450 sample−A450 blank)/(A450 control−A450 blank) × 100

where “sample” characterizes the optical density value of treated cells containing CCK-8 solution and culture media whereas “blank” displays the optical density of CCK-8 solution containing media without cells. IC_50_ values were calculated by SPSS 16.0 version.

### Transfection

A549/T cells were taken for transfection with specific siRNA-MALAT1. The sequence of the sense strand used was MALAT1, 5′-CCC​UCU​AAA​UAA​GGA​AUA​ATT-3′ obtained from GenePharma (Shanghai, China). For transfection, lipofectamine 2000 was used along with siRNA-MALAT1 followed by manufacturer’s protocol. Cells were kept for 4–6 h, later, DMEM was removed and fresh DMEM was added. Transfected cells were used for multiple experiments after 48 h of transfection.

### Extraction of RNA, Synthesis of cDNA, and Quantitative Polymerase Chain Reaction (qPCR)

Total RNAs were extracted and isolated through Trizol reagent (Invitrogen) as guided by the manufacturer’s instructions. For reverse transcription, Prime Script TMRT-reagent kit (TransGen Biotech, China) was used while Trans Start Tip Top Green qPCR Super Mix (TransGen Biotech, China) was used for qPCR according to the instructions given by the manufacturer. The primers sequences were as follows. MALAT1: 5′-GCA​TTA​ATT​GAC​AGC​TGA​CCC​A-3′ (F), 5′-GCT​TGC​TCC​TCA​GTC​CTA​GCT​T-3′ (R); FUT4: 5′-ACAACTGTTCCCGATTCACG-3′(F), 5′-TGCCCTCCTCACCTTTCTC-3′(R); STAT3: 5′-CTT​TGA​GAC​CGA​GGT​GTA​TCA​CC-3′ (F), 5′-GGTCAGCATGTTGTACCACAGG-3′(R); GAPDH: 5′-AGC​CCA​TCA​CCA​TCT​TCC​AG-3′ (F), 5′-ACC​CAT​CAC​AAA​CAT​GGG​GG-3′. GAPDH was used to normalize the data and the relative measurements were determined using the 2^−ΔΔCt^ method. All the experiments were performed independently in triplicate.

### Acquiring Chemical Structures

Chemical structures of ALT and Brv-A were acquired from SciFinder (http://scifinder.cas.org) by using Chem Bio Draw. Afterward, the data was downloaded in the “mol2” format.

### Collection of Compound’s Target and Correction of Protein’s Names

To predict the targets of ALT and Brv-A, Pharm Mapper (http://lilabecust.cn/pharmmapper/), was used that contains a pharmacophore mapping method to identify potential drug targets (Liu et al., 2010). Due to the non-standard names of protein of the extracted targets, the official identifiers were corrected and obtained *via* UniProtKB (http://www.uniprot.org/).

### GO, Pathway Enrichment and Network Construction

DAVID (version 6.8), accessed at https://david-d.ncifcrf.gov, was used for ontologies (GO) and KEGG pathway as mentioned earlier (Sherman and Lempicki, 2009) while Cytoscape program (version 3.4.0) was used to create pathway networks (Missiuro et al., 2009).

### Microscopic Observations of Cellular Morphological Changes

A549/T and A549 cancer cells were added in 96-well culture plates at 37°C overnight and designated treatment of ALT, Brv-A, and PTX was given for 24 h. To determine and photograph the morphological changes in the cells, Phase-contrast microscope (Leica, DMIL LED) was used.

### Live/Dead Assay

To perform live/dead assay, A549/T cells were treated with 5, 10, and 15 µM of ALT and Brv-A for 24 h. After washing the cells with PBS, calcein-AM (2 µM) and PI (4 µM) were added to each sample for 15–20 min and kept at room temperature in the dark. The extra stain was removed by washing with PBS. Later, cells were mixed in PBS, and photographed using fluorescence microscope (Leica, DMI 4000B). To evaluate the live and dead cells, three different fields were selected and 100 cells in each field were calculated.

### Colony Formation Assay

Colony formation assay was done to determine the cytotoxic and antiproliferative effect of ALT and Brv-A. Briefly, A549/T cells were cultured and treated with 5, 10, and 15 µM concentrations of ALT and Brv-A for 24 h. Afterwards, PBS was used to wash the cells, trypsinized, and were seeded in six-well culture plates with cells density of 500 per well. Cells were kept at 37°C for next 7 days to form colonies and given media was changed after each 48 h. After colony formation, cells were washed, and 4% paraformaldehyde (PFA) was used to fix colonies for 15–20 min. Colonies were further stained using crystal violet for 25–30 min. Extra stain was washed by PBS and colonies were photographed. The rate of proliferation was also evaluated by the addition of methanol to dissolve the stain taken by the colonies. Optical density at 595 nm wavelength was determined by fluorescent-spectrophotometer (Synergy neo HTS multi-mode microplate-reader; BioTek).

### Apoptosis Assay

The impact of ALT and Brv-A in the induction of apoptosis was determined through flow cytometry where apoptosis detection kit was used. Annexin V-FITC/PI double staining was performed as directed by the manufacturer’s instruction. In brief, A549/T cells were seeded in six-well culture plates and treated with 5, 10, and 15 µM ALT or Brv-A for 24 h. After washing with PBS, cells were mixed in the provided 1X binding-buffer. Each sample was incorporated with 5 µl annexin V-FITC and 10 µl PI and kept in the dark for 20 min, filtered and subjected to the flow cytometry (Beckmen Coulter, Life Sciences, United States) to investigate apoptotic rate. NovoExpress (version 1.3.0) was used for the analysis of data.

### Cell Migration and Invasion Assay

Anti-migration and anti-invasive capabilities of ALT and Brv-A in A549/T lung cancer cells (transfected with or without siRNA MALAT1) were determined by transwell migration and invasion assays. In brief, cells were added in the transwell chamber without/with Matrigel with density of 3 × 10^3^/chamber. The lower section contained DMEM (10% FBS) as chemoattractant while the DMEM (2% FBS) was poured in the upper chamber. To achieve the rate of migration and invasion among treated and untreated cells, the Transwell chamber was kept at 37°C. Next, the adhesive cells to the inner side of the upper chamber were detached with a soft cotton swab and the cells at the lower side of the chamber were fixed using 4% PFA for 15–20 min. Cells were stained for 30 min through 1% crystal violet, and later PBS was used to wash and remove the extra stain. The chambers were left air dried, photographed by using a fluorescence microscope (Leica, DMI 4000B), and quantified by counting in at least three random fields.

### Protein and Ligand Preparation and Molecular Docking Analysis

To prepare FUT4 and P-GP proteins for molecular docking, dock prep was performed by using USCF Chimera (Pettersen et al., 2004; Yang et al., 2012). ALT and Brv-A were selected for docking purpose and compound’s charges were minimized, and converted into PDBQT format before docking. The partial charges were added and non-polar hydrogen atoms were merged to the ligand atom by PYRX software (Dallakyan and Olson, 2015). Further, docking affinities were carried by selection of predicted ligand binding residues of FUT4 and P-GP proteins. Results were visualized by Discovery Studio software (Dassault Systemes BIOVIA, BIOVIA Discovery Studio Visualizer, v16).

### Protein-Protein Interaction (PPI)

The Search Tool for the Retrieval of Interacting Genes (STRING) was used to evaluate and integrate the protein-protein interactions containing both physical (direct) and functional (indirect) interconnections.

### Western Blot Analysis

Cells, after washing with chilled PBS, were lysed by using RIPA-lysis buffer (Beyotime Biotechnology) carrying 1% PMSF on ice for 30 min. Sodium fluoride (NaF) (2%) was also supplemented to RIPA buffer during extraction of phosphorylated proteins. Later, centrifuged the cells for 15 min at 12,000 rpm and the supernatant was transferred to ice-chilled Eppendorf tubes. The BCA protein assay kit (Beyotime Biotechnology) was used to determine the concentration of protein. Furthermore, by using 10–12% sodium dodecyl sulfate-polyacrylamide gel electrophoresis (SDS-PAGE) gels, an equal amount of 30–40 µg protein was separated, transferred to the PVDF (polyvinylidene difluoride) membranes, and kept in 5% skim milk for 2 h at room temperature. The membranes were further washed with TBST (Tris-buffered saline-tween) three times and incubated in specific primary antibodies overnight at 4°C on a shaker. Next, the membranes were washed by TBST three times and kept in HRP-conjugated goat anti-mouse IgG or goat anti-rabbit IgG secondary antibodies at room temperature for 2 h. After washing by TBST, antibody binding was detected in DNR-bioimaging system MicroChemi 4.2 using ECL plus chemiluminescence kit. GAPDH was internal control for all triplicate experiments performed in the study. ImageJ software was used to analyze the variations in protein expression.

### Statistical Analysis

Among three different performed experiments, all values are depicted as Mean ± SD and compared statistically with untreated, non-resistant, non-transfected samples as control. Student *t-*test was used to compare only two groups while one-way ANOVA followed by Tukey’s Multiple Comparison Test was used for comparison between three or more groups. *p* < 0.05 was considered to be statistically significant.

## Results

### MALAT1 Is Associated With STAT3 Activation and FUT4 Expression in Paclitaxel Resistant Lung Cancer Cells

**FIGURE 1:**
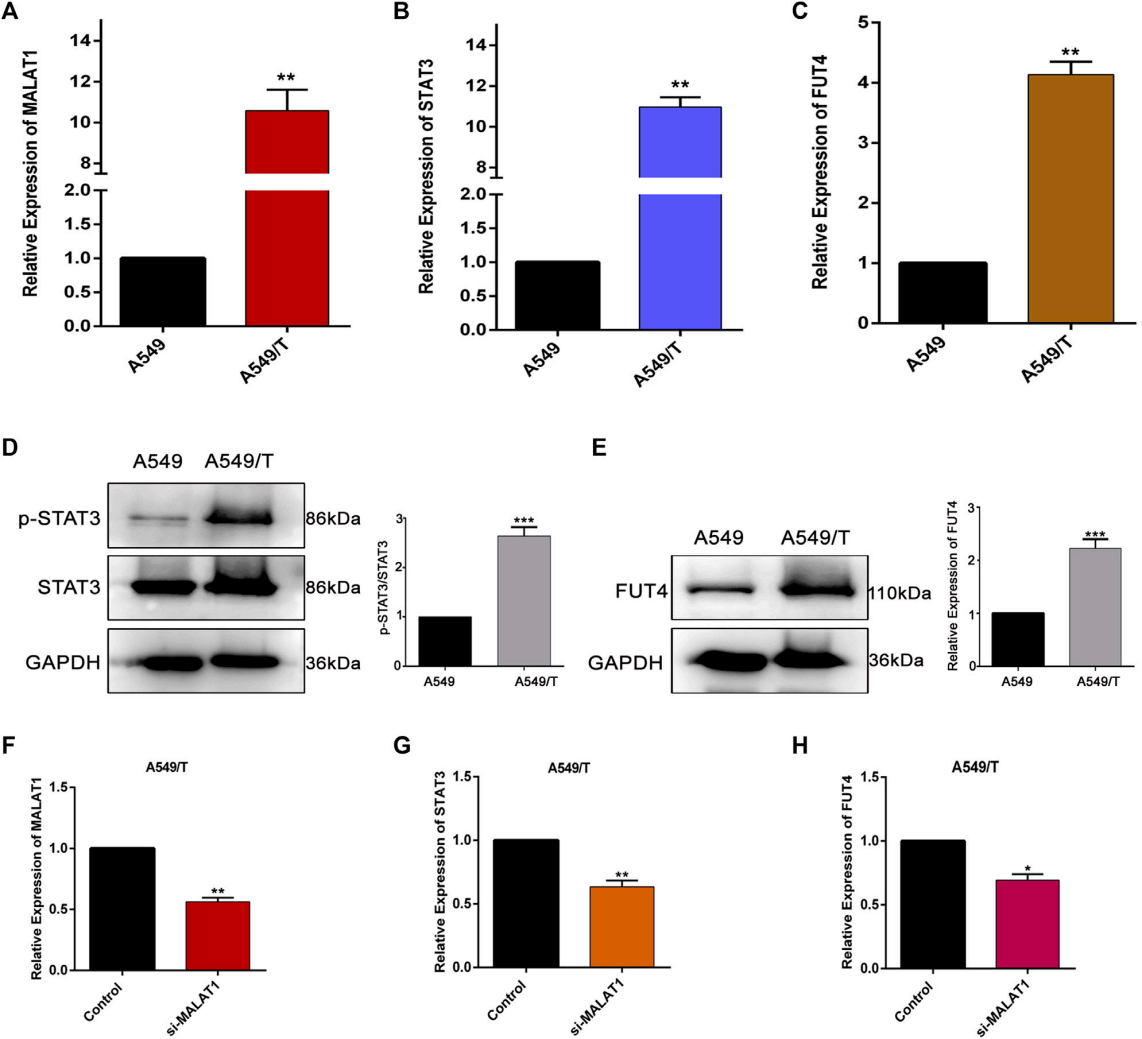
Expression and correlation of MALAT1, STAT3, and FUT4 in A549/T lung cancer cells. **(A)** qPCR analysis for the determination mRNA level of MALAT1 in A549 lung cancer cells compared to that in A549/T cells. **(B)** a comparative qPCR-based analysis of mRNA level of STAT3 among A549/T and A549 lung cancer cells. **(C)** Relative mRNA levels of FUT4 among A549/T and A549 lung cancer cells. **(D)** Comparison of p-STAT3/STAT3 expression among A549/T and A549 cancer cells through western blotting. **(E)** Comparison of FUT4 expression among A549/T and A549 cancer cells through western blotting. **(F)** Functional confirmation of siRNA MALAT1 in inhibiting MALAT1 expression among A549/T cells. **(G)** Expression of STAT3 measured by qPCR in A549/T cells transfected with si-MALAT1 and compared to that in the control. **(H)** Expression of mRNA level of FUT4 measured by qPCR in A549/T cells transfected with si-MALAT1 and compared to that in the control. **(A**–**H)** Data are presented as Mean ± SD, whereas, the entire experiments were done in triplicate independently. ***p < 0.001, **p < 0.01, *p < 0.05 vs control group.

To evaluate the significance of MALAT1, STAT3, and FUT4 in paclitaxel-resistance, initially, we compared the expression levels of lncMALAT1, STAT3, and FUT4 of A549/T cells with non-resistant A549 cells. We quantified the expressions at mRNA and protein level using qPCR and western blotting respectively. As indicated in Figures 1A–C, the mRNA expression of lncMALAT1, STAT3, and FUT4 was significantly high in A549/T cells as compared with the non-resistant A549 lung cancer cells. Similarly, the higher protein level of STAT3 and FUT4 was found in A549/T cells than A549 cells (Figures 1D,E). After establishing the expected role of MALAT1, STAT3, and FUT4 in resistant and non-resistant lung cancer cells, we further studied the association of MALAT1 with STAT3 and FUT4 expression in A549/T cells. Prior to find this association, we first measured the mRNA level for lncMALAT1 in siRNA MALAT1 transfected A549/T resistant cells to establish a control for our further readings. The expression of MALAT1 was downregulated successfully in siRNA MALAT1 transfected A549/T cells (Figure 1F). Later, we assessed the expression of STAT3 and FUT4 in A549/T cells transfected with siRNA-MALAT1. We detected a significant decrease in STAT3 activation and FUT4 expression in resistant cells transfected with siRNA-MALAT1 (Figures 1G,H). Collectively, our data indicate that STAT3 and FUT4 are overexpressed in A549/T cells and this overexpression is associated to the upregulation of lncRNA MALAT1.

### Compound-Target Networking Analysis and Evaluation of Pathways and Gene Ontology (GO)

**FIGURE 2:**
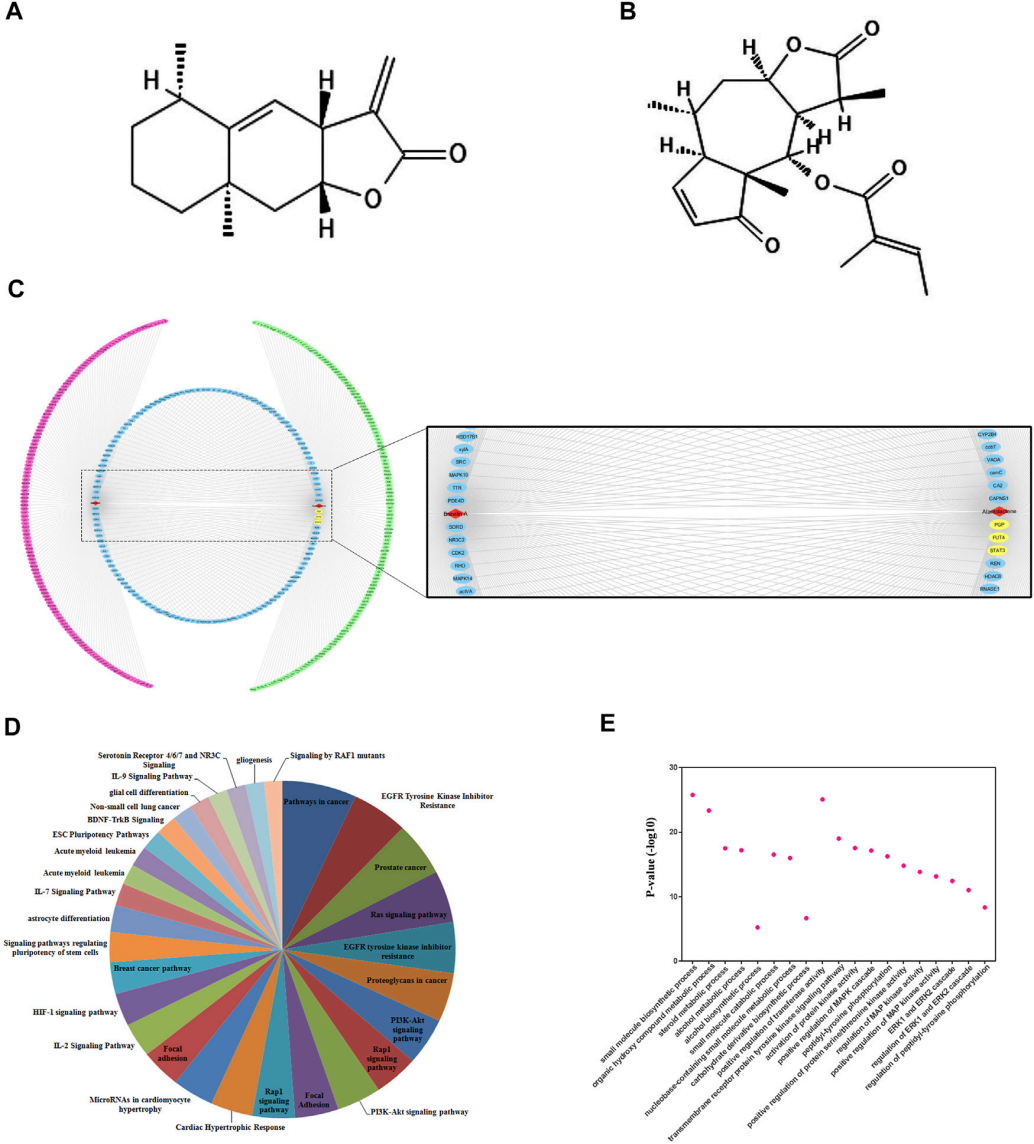
Compound-target networking and evaluation of Gene Ontology (GO) and pathways. **(A)** Chemical structure of Alantolactone **(B)** Chemical structure of Brevilin A **(C)** Red and green circles represent the individual targets of ALT and Brv-A respectively. The blue circle represents some common targets of ALT and Brv-A. ALT and Brv-A are viewed in red triangles while the yellow marked targets are used for further study. **(D)** The *y*-axis depicts *p*-value enrichment score that are greatly enriched, while the names of these enriched biological processes are shown on *x*-axis. **(E)** KEGG Pathways categories.

Prior to the mechanistic study, we validated the targets of SLs by compound-target networking analysis. Among SLs family, we chose ALT and Brv-A (Figures 2A,B) for the current study, as *1*) the anti-cancer effect and their pivotal role in the inhibition of STAT3 signaling has been extensively reported *2*) we confirmed that STAT3, FUT4, and P-GP were the common targets of ALT and Brv-A. There were 536 nodes (two compound nodes and 534 compound-targeted nodes) and 560 edges throughout this network (Figure 2C). The central nodes of this network, 128 nodes in blue, were found to be controlled by both ALT and Brv-A, whereas the external nodes, in green and red, are regulated individually by ALT and Brv-A, respectively. All such controlled nodes could be the core or central nodes in the treatment of lung cancer. As shown in this network, ALT and Brv-A would interact with these targets and exert a significant pharmacological effect.

The potential effect of ALT and Brv-A was further investigated using KEGG pathway evaluation. The analysis represented the enriched pathways in cancer affected by ALT and Brv-A include EGFR-tyrosine kinase inhibitor, PI3k-Akt signaling pathway, RAS signaling pathway, RAP1 signaling pathway, IL-17 signaling pathway, and breast cancer pathway along with EGFR tyrosine kinase inhibitor (Figure 2D). Lung cancer is associated with the aforementioned biological processes. As a result, these findings provided a theoretical support that the anti-cancer activity of ALT and BRV-A is closely connected to the above-mentioned cancer pathways.

We used GO enrichment to further investigate the molecular functions associated with ALT and BRV-A targets. These targets were found not only to be involved into the biosynthesis of small molecules, alcohol metabolism, regulation of ERK1 and ERK2 cascade, but also implicated in regulation of phosphatidyl-tyrosine phosphorylation and the positive regulation of MAPK cascade (Figure 2E).

### ALT and Brv-A Restrict Growth and Proliferation in A549/T NSCLC Cells

**FIGURE 3:**
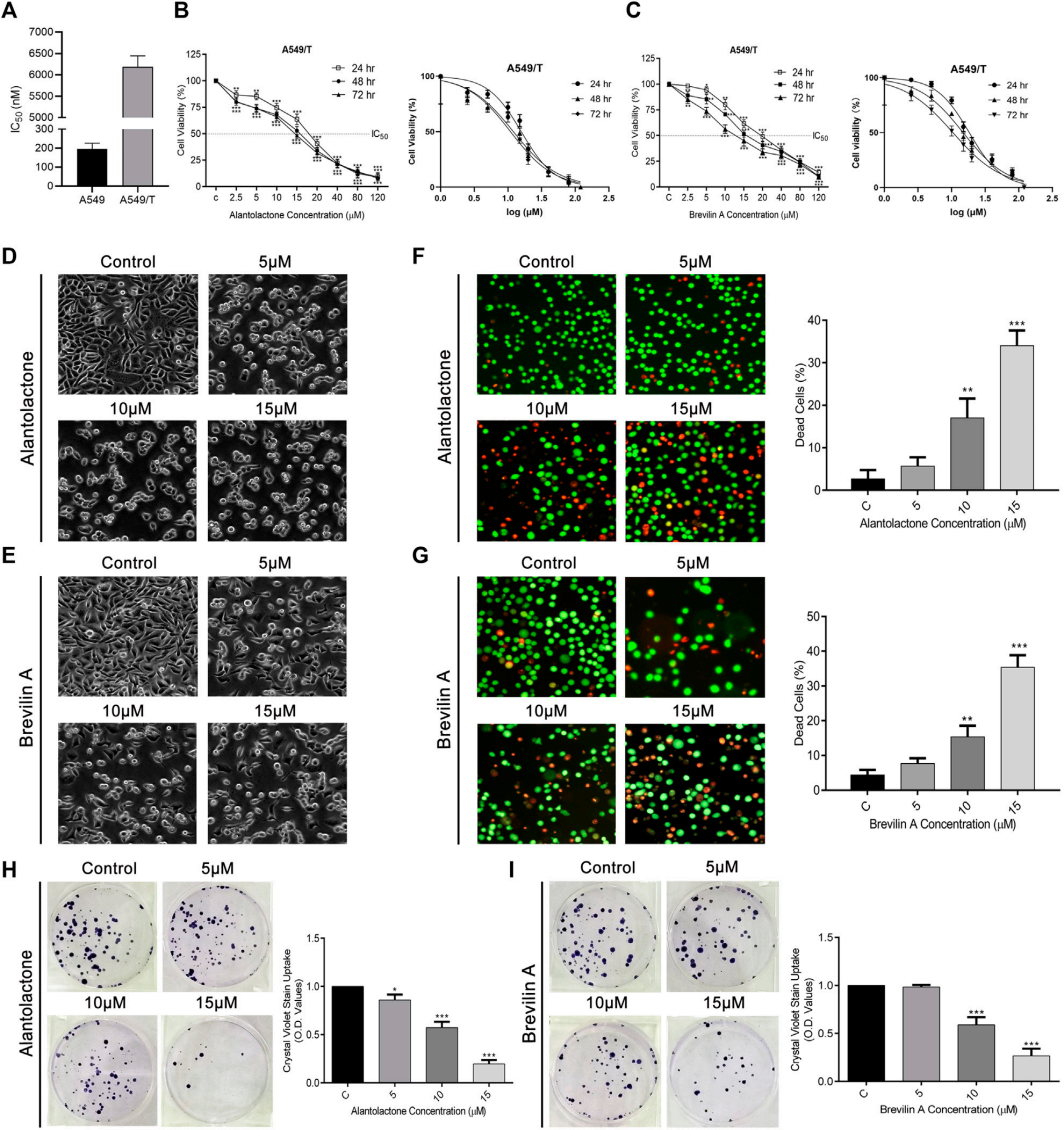
ALT and Brv-A Restrict Growth and Proliferation in A549/T NSCLC Cells. **(A)** Paclitaxel resistance index in A549/T cells compared to the parental A549 cells after treating with various concentrations of paclitaxel for 72 h. **(B)** Cell viability percentage and S curve of A549/T cells treated with different concentrations of ALT (µM) for 24, 48, and 72 h. **(C)** Cell viability percentage and S curve of A549/T cells treated with different concentrations of Brv-A (µM) for 24, 48, and 72 h **(D**,**E)** Morphological changes observed in A549/T cells treated with 5, 10, and 15 µM concentrations of ALT and Brv-A for 24 h **(F**,**G)** Live/dead assay was performed to measure the alive and dead cells percentage by using calcein-AM and PI stain. Cells were treated with 5, 10, and 15 µM concentrations for 24 h and stained with calcein-AM and PI and photographed by using fluorescence microscope (Leica, DMI 4000B). **(H**,**I)** Clonogenic assay was performed to validate the cell viability of A549/T cells. **(D**–**G)** Scale bar is 100 mm. **(A**–**C**,**F**–**I)** Data are presented as Mean ± SD while the entire experiments were done in triplicate independently. ***p < 0.001, **p < 0.01, *p < 0.05 vs control group.

First, we determined the resistance index of paclitaxel among A549 and A549/T cells at 72 h time point. As presented in Figure 3A, the IC_50_ of paclitaxel among A549 cells was approximately 0.195 µM while that in A549/T cells was 6.183 µM on average (almost 31 times higher in A549/T cells) at 72 h indicating that A549/T cells are resistant to paclitaxel. Following that, different concentrations of ALT and Brv-A were used to treat A549/T cells for 24, 48, and 72 h to assess cell viability. We detected a halted cell growth in presence of these two drugs followed by a substantial dose-dependent decrease in A549/T cells viability. The IC_50_ values for ALT were 20, 16, and 15 μM at 24, 48, and 72 h respectively. Moreover, the IC_50_ values for Brv-A at 24, 48, and 72 h were 18.31, 14.84, and 10.45 µM respectively representing the effectiveness of these natural compounds against paclitaxel resistance in lung cancer cells (Figures 3B,C). The most promising concentrations along the suggested concentration gradient of 2.5–120 µM were 5, 10, and 15 µM. The cytotoxic effect of ALT and Brv-A was further examined by observation of cell-morphological changes. Cells, treated with ALT and Brv-A for 24 h, developed a round shape and lost anchorage and cellular geometry (Figures 3D,E). We further confirmed the cell death *via* live/dead assay. PI and Calcein-AM were used to stain the dead (red) and live (green) cells respectively. Results showed that ALT and Brv-A treatment enhanced the proportion of PI intake among A549/T cells in concentration-dependent manner representing the dead cells (Figures 3F,G). Next, we performed colony forming assay to evaluate the anti-proliferative efficacy of ALT and Brv-A. After exposing the A549/T cells to ALT and Brv-A for 24 h, we noted that the capability of resistant cells to form colonies was reduced. We assessed the growth constraints in treated cells by liquifying the crystal violet stain (taken by the colonies) in methanol. Consistent with the reported alterations in cell viability and morphology, we observed lower uptake of crystal violet stain in ALT (Figure 3H) and Brv-A (Figure 3I) treated A549/T cells as compared to untreated cells. The collective data show that ALT and Brv-A has a considerable inhibitory effect over the growth and proliferation rate of A549/T cells.

### ALT and Brv-A Induce Pro-apoptotic Effect in A549/T Cancer Cells

**FIGURE 4:**
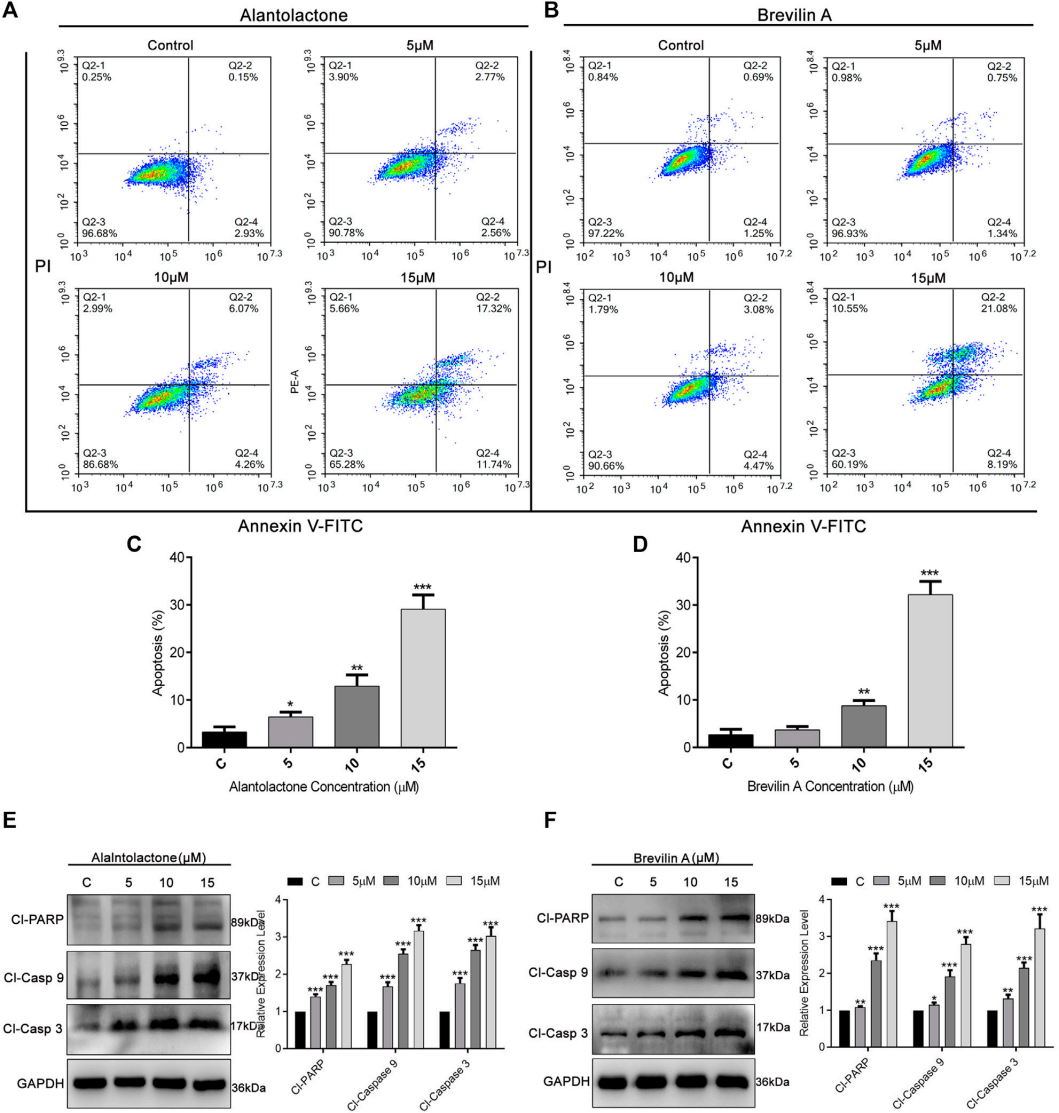
Induction of apoptosis by ALT and Brv-A in A549/T cells. **(A**,**B)** Cells were treated with 5, 10, and 15 µM concentrations of ALT and Brv-A for 24 h. Cell were stained with annexin V-FITC and PI and analyzed by by flow cytometry. **(C**,**D)** Graphical presentation of apoptotic rate (percentage) among A549/T cells treated with ALT and Brv-A for 24 h **(E**,**F)** Expressions of cleaved PARP, cleaved caspase-9, and cleaved caspase-3 were determined by western blotting among treated and untreated A549/T cells. For loading control, GAPDH was used. **(C**–**F)** Data are presented as Mean ± SD while the entire experiments were done in triplicate independently. ***p < 0.001, **p < 0.01, *p < 0.05 vs control group.

To ascertain the mode of cell death, we performed Annexin V-FITC and PI staining and subjected the cells to flow cytometric analysis. As presented in Figures 4A,B, ALT and Brv-A influenced the apoptotic event in dose-dependent way. The percentage of early and late apoptosis (average) was increased from 3.2 to 6.45, 12.9, and 29.08%, after treating the A549/T cells with 5, 10, and 15 µM concentration of ALT respectively (Figure 4C). Similarly, 5, 10, and 15 µM doses of Brv-A increased the rate of apoptosis from 2.65 to 3.68, 8.78, and 32.18% respectively (Figure 4D). Moreover, ALT and Brv-A increased the expression of cleaved PARP, cleaved caspase-9, and cleaved caspase-3 in dose-dependent manner which are the markers of apoptotic processes (Figures 4E,F).

### ALT and Brv-A Inhibit STAT3 Activation and Attenuate FUT4 and P-GP Expression in A549/T Cells

**FIGURE 5:**
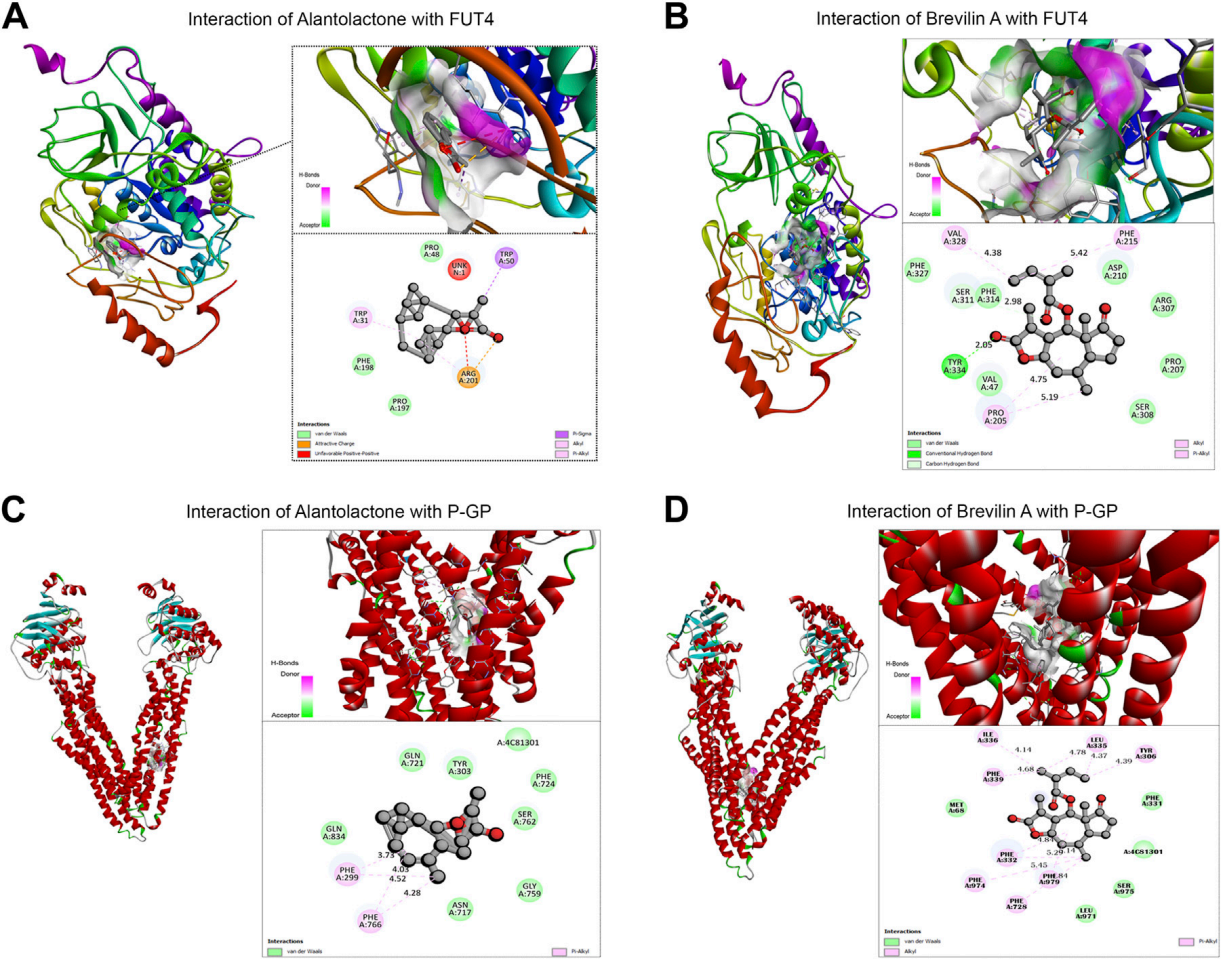
Molecular docking of ALT and Brv-A to determine their binding affinities with FUT4, and P-GP. **(A, B)** Molecular docking of ALT and Brv-A with FUT4. **(C, D)** Molecular docking of ALT and Brv-A with P-GP.

Our previous studies established the direct binding of Brv-A to the STAT3 with a considerable effect compared to commercially available STAT3 inhibitors (S31-201) (Khan et al., 2020). According to the findings of compound networking constructions and previous experimental research regarding anticancer activity of ALT and Brv-A (Maryam et al., 2017; Khan et al., 2020), we chose STAT3, FUT4, and P-GP (with a diverse role in chemoresistance) as the targets. In current study, molecular docking for FUT4 and P-GP was made to assess the effectiveness of primary drug-protein associations and to examine the reliable binding interfaces, though the molecular docking of these two compounds for STAT3 has already been reported in previous studies (Chun et al., 2015; Khan et al., 2020). The molecular docking (protein-ligand complexes analysis) of FUT4 and P-GP with ALT and Brv-A were performed using PyRx software program. First, the validation method was conducted to ensure the capability of docking machine and the RMSD values were ≤2.0 Å. As presented in Figure 5A, the interaction of FUT4 structure with ALT forms a complex *via* carboxylic group of ALT interacted by hydrogen bonding with attractive charged residue Arg-201, pi-alkyl group bonded with residue Trp-31, and pi-sigma interacted to Trp-50 amino acid. As shown in Figure 5B, Brv-A moieties form a complex with FUT4 *via* alkyl and pi-alkyl groups interacting with Phe-215, Val-328, Pro-205, and conventional hydrogen bond to Tyr334. These results showed that FUT4 had the binding affinity of −12.4 Kcal/mol and −8.1 Kcal/mol with ALT and Brv-A respectively. ALT also showed good interaction to P-GP binding site, where hydrogen bond forms interaction between pi-alkyl group and Phe-299 as well as Phe-766 residues (Figure 5C). Brv-A was found to be in interaction with P-GP residues Tyr-306, Phe-332, Leu 335 by alkyl group and pi-alkyl group interacts with amino acids Phe-979, Phe-974, Phe728, Phe-339, and Ile-336 (Figure 5D). On the basis of the binding energy scores of ALT (−10.5 Kcal/mol) and Brv-A (−8.7 Kcal/mol) with P-GP, it is estimated that P-GP has a better binding affinity with ALT.

**FIGURE 6:**
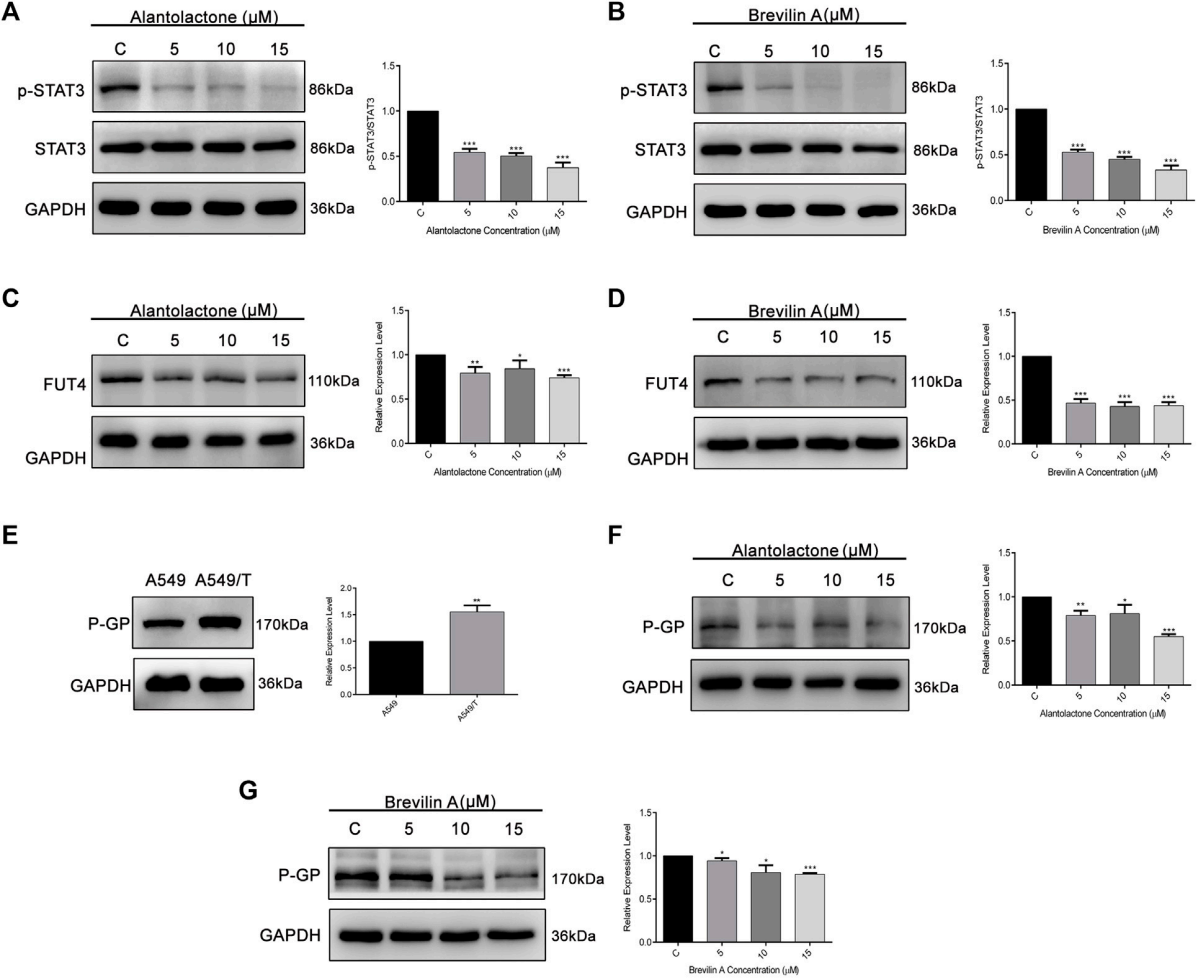
ALT and Brv-A inhibit STAT3 activation, and FUT4 and P-GP expression in A549/T cells. **(A**,**B)** A549/T cells were exposed to the 5, 10, and 15 µM concentrations of ALT and Brv-A for 24 h and cell lysates were prepared. Expression of STAT3 and p-STAT3 was determined through western blotting. **(C**,**D)** Expression of FUT4 was determined through western blotting after treating the cells with 5, 10, and 15 µM concentrations of ALT and Brv-A. **(E)** A comparative expression level of P-GP was determined among A549 and A549/T cells *via* western blotting. **(F**,**G)** Expression of P-GP was determined through western blotting after treating the cells with 5, 10, and 15 µM concentrations of ALT and Brv-A. **(A**–**G)** For loading control, GAPDH was used. Data are presented as Mean ± SD while the entire experiments were done in triplicate independently. ***p < 0.001, **p < 0.01, *p < 0.05 vs control group.

On the basis of previously reported and obtained results from molecular docking, we were interested to analyze the effect of ALT and Brv-A over STAT3 activation, and FUT4 and P-GP expression *via* western blotting. As expected, ALT and Brv-A inhibited activation of STAT3 in A549/T cells in dose-dependent fashion (Figures 6A,B). Furthermore, ALT and Brv-A also inhibited FUT4 expression in dose-dependent manner (Figures 6C,D). P-GP, also known as MDR1, ABCB1 is a transmembrane transporter that works for the efflux of various anticancer drugs across the cell membrane *via* ATP hydrolysis and has been implicated in the promotion of drug resistance in various cancer cells. Additionally, STAT3 plays significant role in transcriptional regulation of P-GP expression (Wang R. et al., 2020). As mentioned in previous studies, targeting P-GP transporter is a viable strategy to overcome paclitaxel resistance in A549 cells (Lin et al., 2020). Therefore, we were interested in determining whether these two SLs members have the capability to downregulate P-GP expression among A549/T cells. To begin, we assessed and compared the expression of P-GP between A549 and A549/T cells. We distinguished a higher expression of P-GP in A549/T cells in contrast to A549 cells confirming the P-GP immersion in paclitaxel resistance (Figure 6E). Next, we determined the expression level of P-GP in A549/T cells treated with 5, 10, and 15 µM concentrations of ALT and Brv-A. Both of these drugs had a dose-dependent inhibitory effect on P-GP expression (Figures 6F,G). The purpose of inhibiting STAT3 activation, P-GP and FUT4 expressions in A549/T cells was to overcome the resistivity, as these proteins are considered to be the major contributors in developing preventive strategies by cancer cells against paclitaxel. Our study shows that ALT and Brv-A has a potential inhibitory effect over STAT3 activation, FUT4, and P-GP all of which are known to be linked to the drug resistance in cancer.

### MALAT1 Inhibition Augments the Cytotoxic, Anti-migratory, and Anti-invasive Competence of ALT and Brv-A in A549/T Cells

**FIGURE 7:**
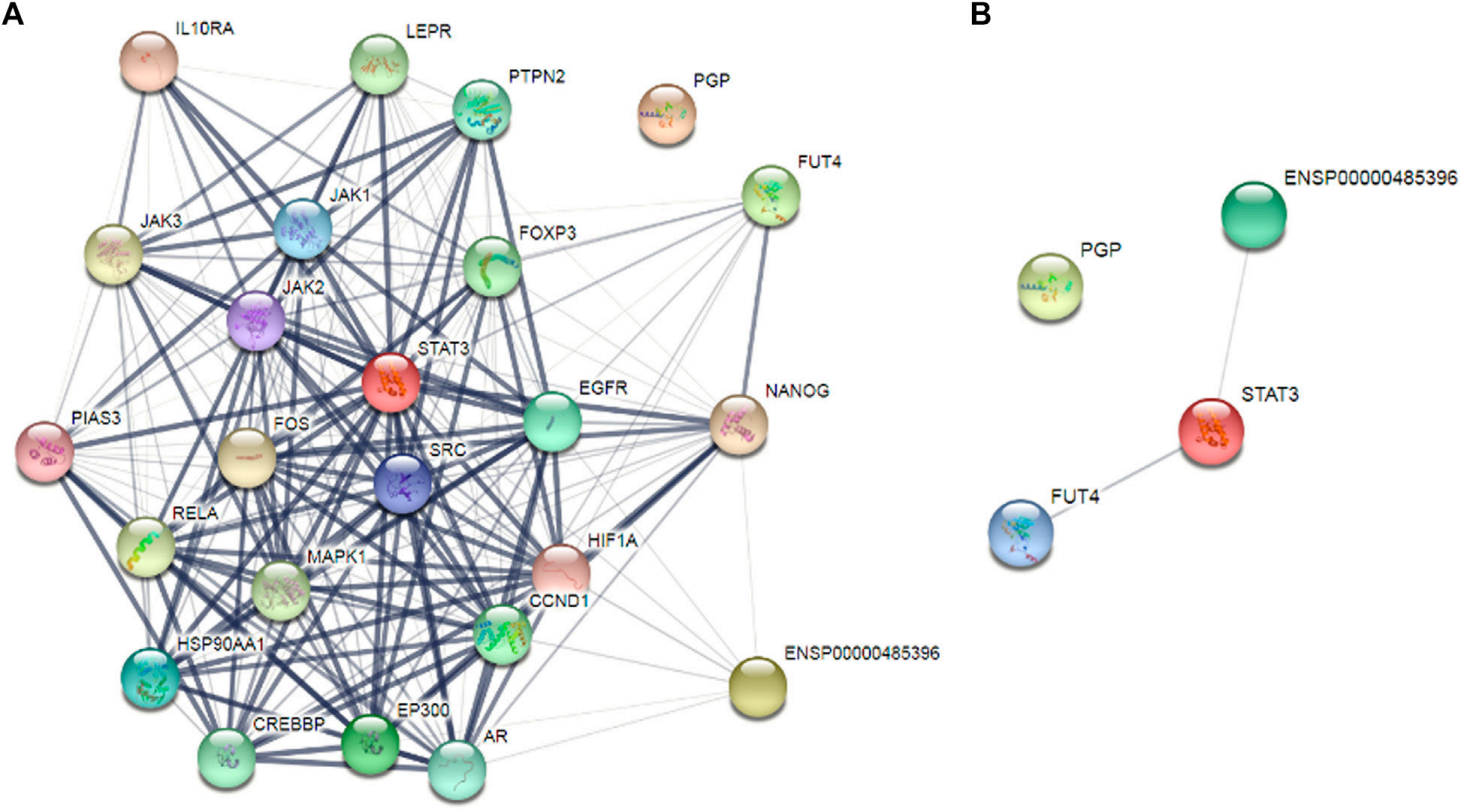
Determination of the interrelating proteins through protein-protein interaction (PPI) network *via* the STRING online tool. **(A**,**B)** Lines in the network represent interaction of proteins among each other.

**FIGURE 8:**
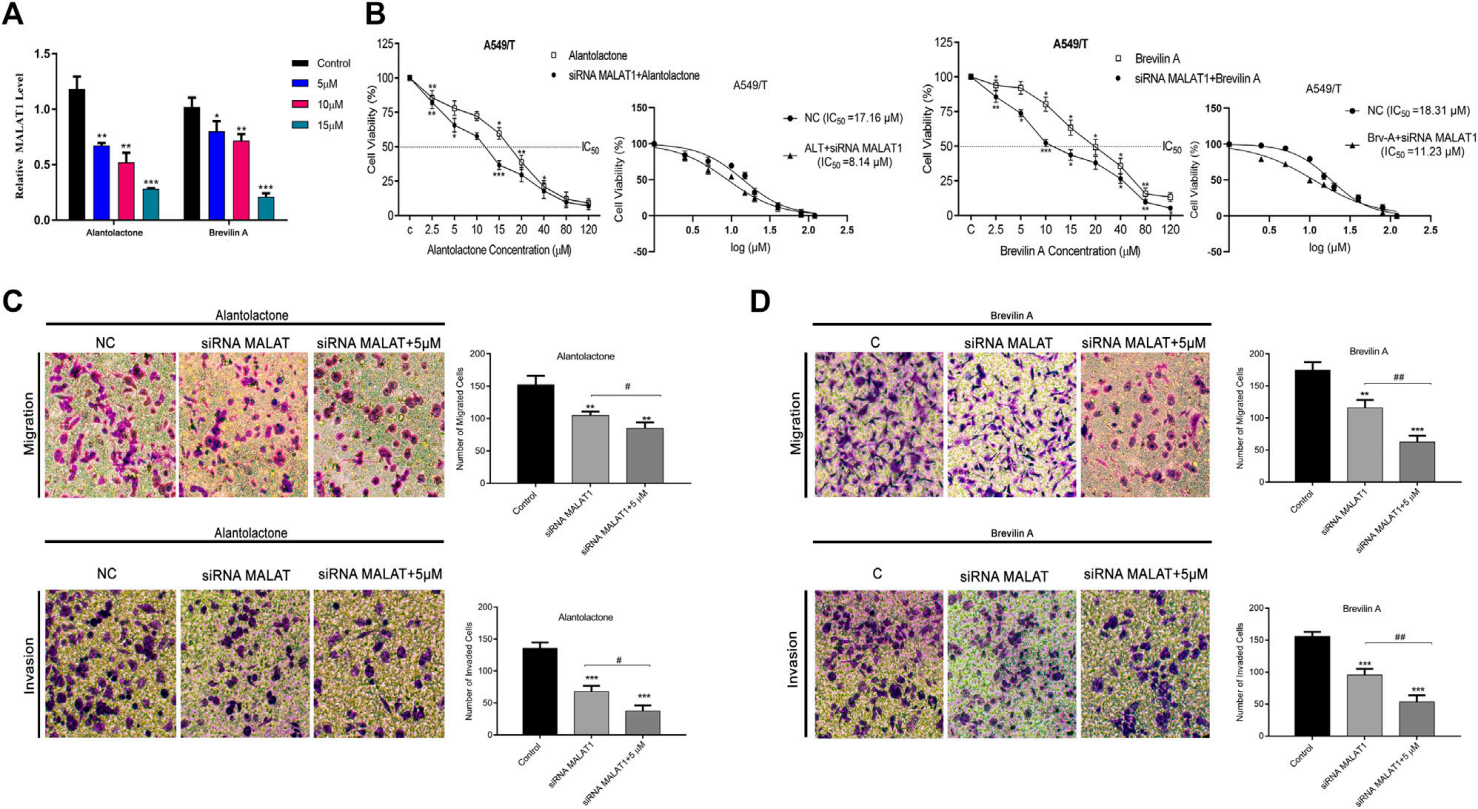
ALT and Brv-A reduce MALAT1 expression to overcome paclitaxel-resistance in lung cancer cells. **(A)** The mRNA level of MALAT1 was measured in A549/T cells treated with 5, 10, and 15 µM concentrations of ALT and Brv-A for 24 h. **(B)** Cell viability (percentage) and S curve of non-transfected and siRNA MALAT1 transfected A549/T cells treated with ALT and Brv-A for 24 h **(C**,**D)** Rate of migration/invasion was measured in siRNA MALAT1 transfected A549/T cells individually and in combination with treatment of ALT and Brv-A (5 µM each). **(A–D)** Data are presented as Mean ± SD while the entire experiments were done in triplicate independently. ***p < 0.001, **p < 0.01, *p < 0.05 vs control group.

After targeting three important modules responsible for paclitaxel drug resistance in cancer i.e., STAT3, FUT4, and P-GP, we were interested to determine the role of MALAT1 in this scenario. Prior to establish the effectiveness of ALT and Brv-A over the functional role of MALAT1, we predicted the protein-protein interactions (PPIs) by using STRING database. Our analysis revealed that MALAT1 interacts with 20 different types of proteins which included FUT4, STAT3, JAK1, JAK2, JAK3, EGFR, MAPK1, FOXP3, FOS, LEPR, PTPN2, HIF-1α, PIAS3, RELA, HSP90AA1, EP300, AR, CREBBP, IL10RA, and NANOG (Figure 7A). As given in Figure 7B, it is estimated that MALAT1 may interact with FUT4 *via* STAT3 that needs further experimental validation. Moreover, previous research has established that MALAT1 modulates STAT3, FUT4, and P-GP (Cheng et al., 2013; Fang et al., 2018; Yang et al., 2018; Wang R. et al., 2020; Xu et al., 2020). On the basis of MALAT1/STAT3, MALAT1/FUT4 and MALAT1/STAT3/P-GP interactions, we hypothesized that ALT and Brv-A may influence the expression of MALAT1. We assessed the MALAT1 levels in drug treated (ALT, Brv-A) groups and compared to the untreated and found a significant decrease in dose-dependent fashion (Figure 8A). Due to strong interactive properties of MALAT1 to STAT3, FUT4, and P-GP (selected markers to be targeted in current study) we were interested to investigate the impact of knockdown MALAT1 over further improving the efficacy of ALT and Brv-A. This was accomplished by determining and comparing the viability of A549/T cells treated with different concentrations of ALT and Brv-A with and without siRNA MALAT1 transfection. As presented in Figure 8B, siRNA MALAT1 transfected cells were more sensitive to ALT and Brv-A at various dosages. Previously, some studies have confirmed the role of MALAT1 in the migration and invasive properties of various cancer cells (Chen et al., 2018; Tang et al., 2018; Yuan et al., 2019). Our data suggested that the inhibition of MALAT1 restricts A549/T cells from migration and invasion individually as well as in combination with treatment of ALT and Brv-A (Figures 8C,D). These results suggested that targeting the STAT3, FUT4, and P-GP and MALAT1 (by ALT and Brv-A as well as *via* gene knockdown) reduce the migration and invasion of paclitaxel-resistant A549 cells and enhance the competence of these two compounds significantly. These findings propose that along with targeting STAT3, FUT4, and P-GP transporters, inhibiting MALAT1 response would promote the rate of apoptosis in drug resistant cancer cells.

## Discussion

Metastasis and chemoresistance are the primary contributors for the terminal condition of lung cancer. Paclitaxel resistance is the major concern for researchers in field of therapeutic tactic towards NSCLC (Lin et al., 2020). Although, recent advancements in therapeutics have made it possible to overcome EGFR-TKIs resistant cancer types, however, the adverse effects, unmapped targets and cellular responses to the available drugs require further investigation (Yuan H. et al., 2016; Chen et al., 2017; Chen et al., 2019). More precisely, because paclitaxel resistance is multifactorial, it likely has various unknown motives that need further amplification with special emphasis on discovery of novel natural compounds with fewer side effects to counter the unexplored therapeutic targets and crucial aspects of resistance.

Several lncRNAs have been described to cause drug resistance in different cancer types which indicates that these lncRNAs serve as biological targets of drug resistance in cancer (Xue et al., 2018). LncRNA MALAT1 enhances tumor proliferation, migration, invasion, and growth of cancer cells. MALAT1 was known as a metastasis development marker for lung adenocarcinoma in the early stages (Ji et al., 2003). MALAT1 has been shown to contribute in a variety of functions in cancer cells such as, enhancement of EMT, acting as miRNA sponges, and stimulating autophagic events (Lin et al., 2007; Ji et al., 2013; Fan et al., 2014; Hirata et al., 2015; Qi et al., 2016). Recently, a numeral study has explored the fundamental role of MALAT1 in the development of drug resistance in different cancer cells. Some of these include resistance to the hepatocellular carcinoma cells by sponging miR-216b (Yuan P. et al., 2016), sunitinib-resistance by regulating miR-362-3p-mediated G3BP1 in renal cell carcinoma (Wang Z. et al., 2020), cisplatin resistance in gastric cancer (Dai et al., 2020), and docetaxel resistance of prostate cancer cells *via* miR-145-5p-mediated regulation of AKAP12 (Xue et al., 2018).

STAT3 is a transcriptional regulator of numerous factors that are involved in tumor progression. STAT3 activation has been shown to promote cancer proliferation, growth, and reduces the apoptotic rate (Timofeeva et al., 2013). Over-activation of STAT3 is linked with resistance to several drugs and therapeutic agents including, crizotinib, erlotinib, gefitinib, trastuzumab, and sorafenib (Sen et al., 2012; Li et al., 2013; Wu et al., 2013; Cuyàs et al., 2016; Aghazadeh and Yazdanparast, 2017; Sakurai et al., 2017). Additionally, alterations in the expression of fucosyltransferases, i.e., FUT4, FUT6, and FUT8 are also associated with MDR resistance (Cheng et al., 2013). Therefore, STAT3 along with FUT family proteins are attractive targets for overcoming drug resistance in cancer. In this study, we confirmed that MALAT1, STAT3, FUT4, and P-GP were higher in A549/T cells as compare to A549 cells. To validate whether MALAT1 is associated with STAT3 activation and FUT4 fucosylation, we knocked down MALAT1 *via* siRNA MALAT1 and determined the STAT3 activation and FUT4 expression. Interestingly, we found that MALAT1 knocked down in A549/T cells resulted in decreased FUT4 expression and STAT3 activation. Our findings were parallel to the findings of previous studies where MALAT1 promoted drug resistance in cancer cells *via* modulating STAT3 activation in lung cancer and enhanced FUT4 fucosylation by sponging miR-26a/26b involving PI3K/AKT pathway (Xu et al., 2020).

In the current study, we were interested in evaluating the anticancer activity of ALT and Brv-A against paclitaxel-resistant lung cancer cells. The aim was also extended to gain insight into whether simultaneous inhibition of STAT3 activation and FUT4 fucosylation *via* ALT and Brv-A have any impact on functional role of lncRNA MALAT1 in A549/T lung cancer cells. Prior to the mechanistic study, we also confirmed the targets of these two compounds by compound-target networking analysis where we found that STAT3, FUT4, and P-GP were common targets for both, ALT and Brv-A. The KEGG pathway analysis further justified the dominant involvement of these compounds in cancer pathways and EGFR tyrosine kinase inhibitor resistance. Therefore, these two natural compounds among SLs family were found more potent for this study. The results indicated that ALT and Brv-A significantly inhibited proliferation, growth, and cell viability of A549/T cells, accompanied by induction of apoptosis in a dose-dependent mode. Further study confirmed the apoptosis *via* measuring expression levels of apoptotic markers, i.e., PARP, caspase-9 and caspase-3 cleavage. Flow cytometric analysis was consistent with the finding where A549/T cells exhibited a significantly higher apoptotic rate than the untreated control group. Our results in terms of inducing apoptosis by ALT and Brv-A were consistent with previously conducted studies where ALT induced apoptosis in squamous lung cancer SK-MES-1 cells (Zhao et al., 2015) as well as in A549 lung adenocarcinoma cells (Maryam et al., 2017) while Brv-A induced apoptosis in breast cancer cells (MCF-7) through generation of ROS (Saleem et al., 2020) and lung cancer cells (Khan et al., 2020).

ALT and Brv-A are well-known natural SLs that inhibit STAT3 activation, while the inhibitory effect over FUT4 has been investigated for the first time in the current study. Moreover, the effect of ALT over P-GP has been investigated in the previous study (Maryam et al., 2017) however, Brv-A is used in the current study to explore its effectiveness against P-GP transporters that play a pivotal role in resistance. Our data confirmed that ALT and Brv-A, effectively inhibited STAT3 activation and decreased FUT4 and P-GP expression in A549/T cells in a dose-dependent manner. We also verified the primary drug-protein associations and reliable binding interfaces *via* molecular docking. The capability to restrict STAT3 activation and FUT4 and P-GP expressions simultaneously in A549/T cells enlist these two natural compounds as a comprehensive therapeutic strategy for incapacitating paclitaxel-resistance.

We also aimed to explore the effect of ALT and Brv-A over MALAT1 expression after exploring their effectiveness against STAT3 activation, FUT4, and P-GP expression. Interestingly, we found restricted MALAT1 expression in A549/T cells treated with ALT and Brv-A. To confirm whether restricting MALAT1 expression may affect the functional role of MALAT1 in paclitaxel resistance, we used siRNA-MALAT1 to knockdown MALAT1 and measured the viability of A549/T cells compared to the negative control. We observed a significant reduction in cell viability of MALAT1 knocked down cells treated with ALT and Brv-A. Inhibition of MALAT1 activity further promoted the effectiveness of these SLs. Our results were similar to the findings of Yang et al., where they showed that knockout of MALAT1 *via* shRNA MALAT1 improved the apoptotic rate of Polyphyllin I promoted in gefitinib-resistant NSCLC (Yang et al., 2018). Next, we were interested to know the role of MALAT1 in promoting invasion and migration of A549/T cells. Restriction of MALAT1 activity at the cellular level attenuated the migratory and invasive competence of A549/T cells. Furthermore, we also explored that ALT and Brv-A in combination with MALAT1 inhibition significantly reduced the migration/invasion among A549/T cells that further justified the role of MALAT1 in paclitaxel resistance and vindicated the rationale of the study.

**FIGURE 9:**
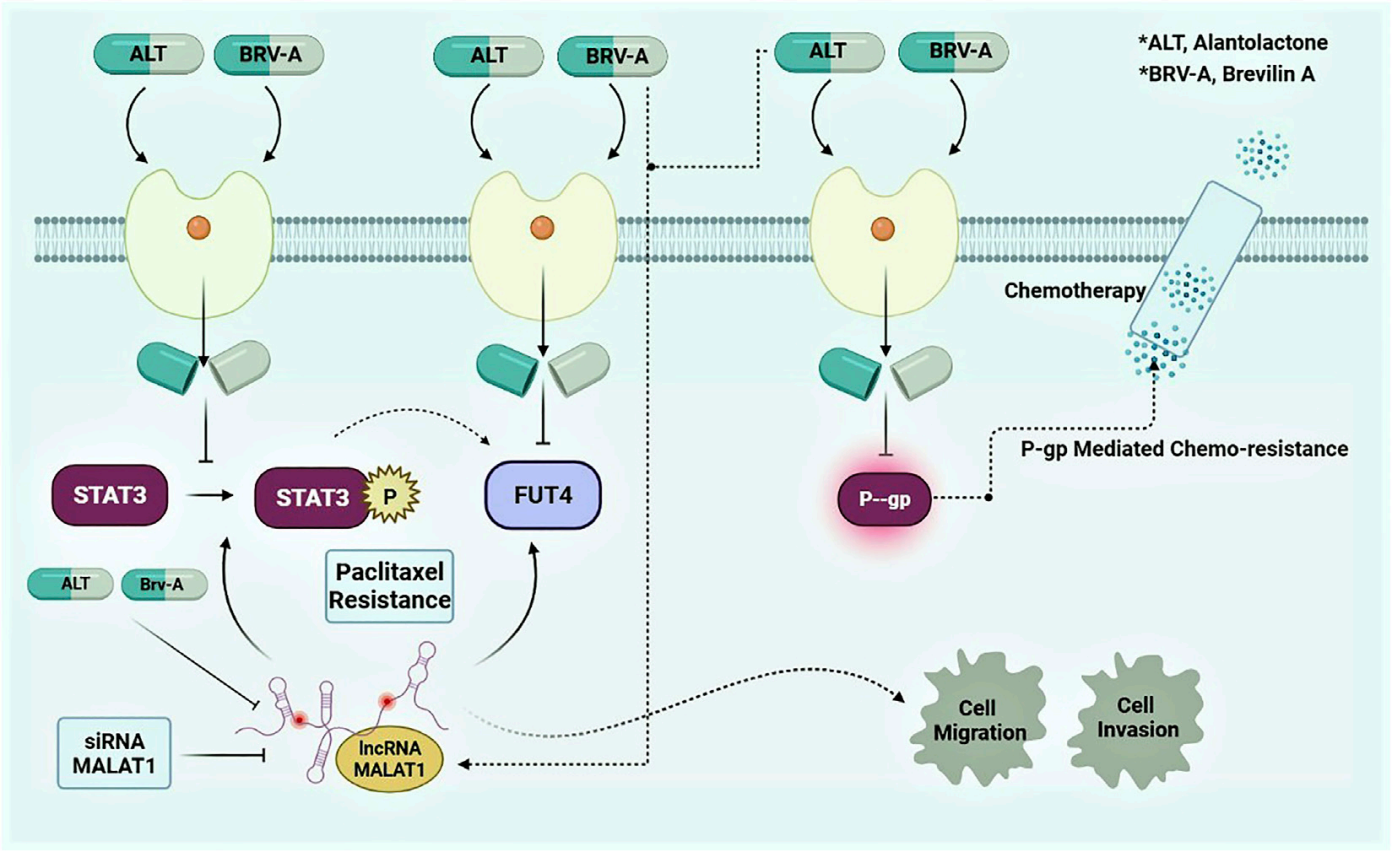
Graphical presentation of mode of action of ALT and Brv-A and preemptive role of MALAT1 in A549/T lung cancer cells.

In conclusion, the higher expression of lncRNA MALAT1 among resistant cancer types is alarming. The potential chemotherapeutic targets regarding chemoresistance e.g., STAT3, FUT4, and P-GP transporters are current center of attention for researcher to deal with drug resistance. It has been considered to be an inclusive strategy tactic for the management of drug resistance in cancer. This study concluded that MALAT1 is the main responsible factor that contributes to the paclitaxel resistance in A549 cells. Moreover, inhibiting STAT3 activation and FUT4 expression along with P-GP *via* SL, the widely used natural compounds for research and therapeutic purpose, demonstrated to overcome drug resistance (Figure 9; graphical presentation). In addition, restricting MALAT1 activity at cellular level could result in improvement in the promotion of apoptosis and prevent migration and invasion of paclitaxel-resistant lung cancer cells. Current study suggests these two SLs to be the promising agents for overcoming paclitaxel resistance in A549 lung cancer cells *via* MALAT1/STAT3/FUT4 axis. The profound insights at molecular level i.e., correlation of the physiological mechanism of MALAT1, STAT3, FUT4 and P-GP are lacking that needs further experimental investigations. However, the given findings suggest improved treatment strategies for patients with paclitaxel-resistant lung cancer with special emphasis over SLs.

## Data Availability

The original contributions presented in the study are included in the article/Supplementary Material, further inquiries can be directed to the corresponding authors.
