# Corrigendum: Sesquiterpene lactones attenuate paclitaxel resistance via inhibiting MALAT1/STAT3/ FUT4 axis and P-Glycoprotein transporters in lung cancer cells

**DOI:** 10.3389/fphar.2024.1363218

**Published:** 2024-04-18

**Authors:** Yaming Ding, Zhang Zhen, Muhammad Azhar Nisar, Farman Ali, Riaz Ud Din, Muhammad Khan, Tafail Akbar Mughal, Gulzar Alam, Linlin Liu, Muhammad Zubair Saleem

**Affiliations:** ^1^ The Second Hospital of Jilin University, Changchun, China; ^2^ College of Basic Medical Sciences, Dalian Medical University, Dalian, China; ^3^ Academy of Integrative Medicine, Fujian University of Traditional Chinese Medicine, Fuzhou, China; ^4^ Institute of Zoology, University of the Punjab, Lahore, Pakistan; ^5^ Medical Toxicology Laboratory, Department of Zoology, Women University of Azad Jammu and Kashmir, Muzaffarabad, Pakistan; ^6^ Faculty of Rehabilitation and Allied Health Sciences, Riphah International University, Islamabad, Pakistan; ^7^ Fujian Provincial Key Laboratory of Natural Medicine Pharmacology, School of Pharmacy, Fujian Medical University, Fuzhou, China

**Keywords:** paclitaxel resistance, STAT3, FUT4, P-gp, MALAT1, alantolactone, brevilin A

In the published article, there was an error in Figures 5B,C as published. The striatum of Figures 5B,C in the article was not the final version. This may have been caused by an initial error in the image layout editing process. The molecular docking presentation in Figure 5B is duplicate of Figure 5A while Figure 5C is pasted incorrectly, however, the description of Figure 5 in the article is correct**.** The corrected Figure 5 and its caption appear below.

**FIGURE 5:**
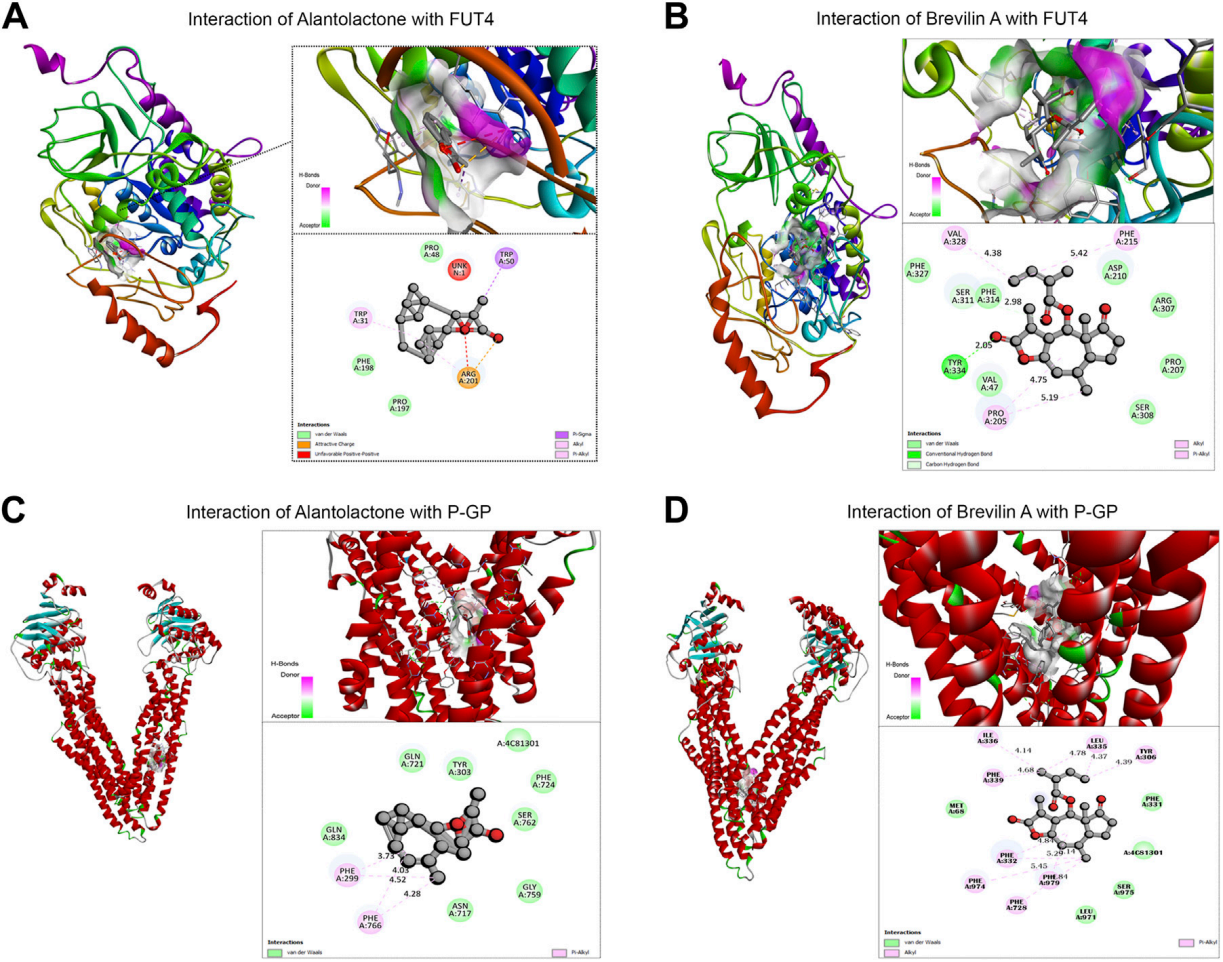
Molecular docking of ALT and Brv-A to determine their binding affinities with FUT4, and P-GP. **(A, B)** Molecular docking of ALT and Brv-A with FUT4. **(C, D)** Molecular docking of ALT and Brv-A with P-GP.

The authors apologize for this error and state that this does not change the scientific conclusions of the article in any way. The original article has been updated.

